# SARS-CoV-2 infection in asymptomatic healthcare workers at a clinic in Chile

**DOI:** 10.1371/journal.pone.0245913

**Published:** 2021-01-28

**Authors:** Claudio Olmos, Gonzalo Campaña, Victor Monreal, Paola Pidal, Nannet Sanchez, Constanza Airola, Dayan Sanhueza, Patricio Tapia, Ana María Muñoz, Felipe Corvalan, Sebastian Hurtado, Claudio Meneses, Ariel Orellana, Martin Montecino, Gloria Arriagada, Fernando Jose Bustos

**Affiliations:** 1 Clinica INDISA, Santiago, Chile; 2 Facultad de Medicina, Universidad Andres Bello, Santiago, Chile; 3 Facultad de Ciencias de la Vida, Centro de Biotecnología Vegetal, Universidad Andres Bello, Santiago, Chile; 4 FONDAP Center for Genome Regulation, Santiago, Chile; 5 Instituto de Ciencias Biomedicas, Facultad de Medicina y Facultad de Ciencias de la Vida, Universidad Andres Bello, Santiago, Chile; BronxCare Health System, Affiliated with Icahn School of Medicine at Mount Sinai, UNITED STATES

## Abstract

Asymptomatic SARS-CoV-2 infection of healthcare workers (HCWs) has been reported as a key player in the nosocomial spreading of COVID-19. Early detection of infected HCWs can prevent spreading of the virus in hospitals among HCWs and patients. We conducted a cross-sectional study to determine the asymptomatic infection of HCWs in a private clinic in the city of Santiago, Chile. Our study was conducted during a period of 5 weeks at the peak of transmission of SARS-CoV-2 in Chile. Nasopharyngeal samples were obtained from 413 HCWs and tested for the presence of SARS-CoV-2 using RT-qPCR. We found that a 3.14% of HCWs were positive for the presence of SARS-CoV-2 (14/413). Out of these, 7/14 were completely asymptomatic and did not develop symptoms within 3 weeks of testing. Sequencing of viral genomes showed the predominance of the GR clade; however, sequence comparison demonstrated numerous genetic differences among them suggesting community infection as the main focus of transmission among HCWs. Our study demonstrates that the protocols applied to protect HCWs and patients have been effective as no infection clusters due to asymptomatic carriers were found in the clinic. Together, these data suggest that infection with SARS-CoV-2 among HCWs of this health center is not nosocomial.

## Introduction

SARS-CoV-2 originated in Wuhan, China, presumably in a food market. Since then it has spread widely around the world, leaving more than 53.5 million positive cases and 1.3 million deaths (WHO) as of November 2020. The first case of COVID-19 in Chile was reported in March 3^rd^, 2020 and from that point most of the country has had cases of SARS-CoV-2 infection leaving more than 450000 cases and 15000 deaths (Chilean Government official data).

COVID-19 presents in patients with fever, cough and chest discomfort that frequently evolves into pneumonia [1]. Nowadays it is known that COVID-19 can also present with minimal or no symptoms at all, which can significantly influence silent viral transmission among the population via droplets, direct contact or fomites [2]. It is estimated that at least 50% of viral transmission among the population is accounted for by asymptomatic or pre-symptomatic individuals [3]. Hence, active screening and isolation of these individuals is critical for stopping viral transmission [4]. In this context, the role that nosocomial transmission of SARS-CoV-2 can have among patients has become relevant, especially in health care workers (HCWs) [5]

Healthcare workers are a high-risk population for viral transmission, particularly because social distancing is not always possible while caring for patients. This is despite all the efforts to provide adequate personal protection elements (PPE) and for establishing protocols to limit SARS-CoV-2 transmission in the workplaces [6]. Previous studies show conflicting evidence suggesting that nosocomial transmission between HCWs could reach up to 44% [7] or 89% [8, 9], while other studies show that HCWs are mainly infected through contacts within their communities outside of their working places [10].

Here we investigate asymptomatic infection of HCWs in a private clinic in the city of Santiago, Chile. Our study was conducted during the peak of transmission of SARS-CoV-2 in Chile and aims to assess whether the correct use of PPE and the infection control protocols established at most health institutions are sufficient to control nosocomial transmission of SARS-CoV-2.

A total number of 413 asymptomatic HCWs were enrolled into the final study. Only 14 tested positive for the presence of SARS-CoV-2 by RT-qPCR, representing a 3.4% of positivity in asymptomatic HCWs. Importantly, our results show that there is no relationship between the SARS-CoV-2 PCR Ct values or viral load of an individual and the subsequent development of any symptoms related to COVID-19. Moreover, sequencing of the viral genomes confirms that there are at least 5 different infection sources among the infected individuals, suggesting that these HCWs became infected at their individual communities outside of the clinic.

## Material and methods

### Volunteer enrollment protocol

All HCWs from Clinica Indisa that had not been infected by SARS-CoV-2 previously and did not present any symptoms at the time of enrollment were able to join the study. Public announcements in the clinic were posted to recruit volunteers. Enrollment to our study was completely voluntary. We were able to reach ~30% of the total HCWs in the clinic. (413/1379). Before sampling, an online questionnaire was applied to all asymptomatic subjects to record: Sex, age, profession, residence commune, comorbidities (chronic respiratory, cardiovascular, rheumatic diseases, others), and other variables related to their occupation as a service in which they work, and exposition. Also, written consent of every enrolled HCW was obtained. This study was approved by the Medical Director of INDISA Clinic and the Scientific Ethical Committee of Universidad Andres Bello.

### Sample collection

Nasopharyngeal swabs were obtained from enrolled subjects and kept in viral transport media at 4°C until processing, following the protocols established by the World Health Organization and the Chilean Public Health Institute (ISP). Samples were collected in the morning and results were informed in the following 8–10 hours. This rapid processing of samples allowed efficient detection of new asymptomatic cases, thereby drastically reducing any potential nosocomial transmission by the subjects. HCWs did not return to work until the result from their qPCR test was informed and was negative for the presence of SARS-CoV-2.

### Viral genome purification and quantification

For isolation of viral genomes, we made use of a Kingfisher Duo Prime automated system using the MagMAX Viral/pathogen nucleic acid isolation kit (Thermofisher, USA), following manufacturer’s instructions. Briefly, 200 μL were taken from nasopharyngeal swabs and mixed with 5 μL of proteinase K solution and 275 μL of binding solution/magnetic beads mix. Samples were incubated at room temperature for 15 minutes, inside a laminar flow hood, to inactivate any viral particles. After incubation, samples were transfer to the Kingfisher Duo Prime system and the protocol was run for 25 minutes, which included 3 washes and then the RNA was eluted in 50μL with elution buffer. To quantify the viral genomes in the samples, the LigthMIX SARS-COV-2 RdRP Roche kit was used using a Roche Lightcycler 480 II PCR machine, following manufacturer’s instructions. Briefly, 5 μL of eluted samples (2-10ng/μL) were used as template for RT-PCR reactions together with a master mix and a set of primers targeting de RdRP gene. An incubation for 5 min at 55°C was performed for RT reaction to proceed. 45 PCR cycles were carried out to amplify SARS-CoV2 genomes. SARS-CoV-2 PCR Ct values <39 were considered positive for the presence of SARS-CoV-2 as indicated by the manufacturer. All HCWs positive in our study were re-tested by an official government licensed laboratory.

### Genome sequencing

We performed the reverse transcription and PCR based on information provided by the Artic Network initiative (https://artic.network/) to obtain complementary DNA and sequencing libraries using the TruSeq DNA Sample Prep kit (illumina Inc.). All samples were sequenced on the Illumina MiSeq platform using the 500-cycle MiSeq Reagent Kit v2 Nano (Illumina) at Universidad Andres Bello. All genomic data from sequenced samples were deposited in NCBI with the following NCBI ID Seq: SAMN16255338, SAMN16255337, SAMN16255336, SAMN16255335, SAMN16255334, SAMN16255333, SAMN16255332, and SAMN16255331.

### Sequencing data analysis

We checked the quality of the raw reads using Fastqc v0.11.8, and then, the reads were filtered and trimmed using Trim_galore v0.5.0. We discarded the reads with a Phred score less than 30 and a minimum read length of less than 50bp. We assembled the SARS‐CoV‐2 genomes by IRMA v0.9.3 using as reference NCBI sequence ID NC_045512.2, and we performed the sequence alignment using MAFFT [11]. We constructed the phylogenetic tree by IQ‐TREE v1.6.12, considering a bootstrap of 1,000 iterations. We used our eight assembled genomes and 162 full-genomes available in GISAID (https://www.gisaid.org/).

## Results

**Fig 1:**
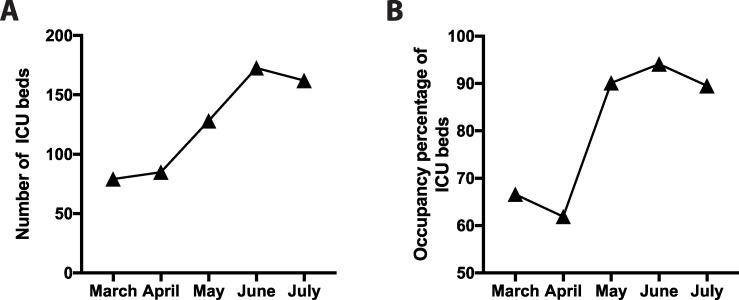
Capacity and occupancy of ICU bed in Clinica Indisa. (A) Total number and (B) occupancy percentage of ICU beds between March and July 2020; the peak transmission times of SARS-CoV-2 in Santiago, Chile.

We aimed to screen the HCW population that were in direct contact with COVID-19 patients at Clinica Indisa in Santiago, Chile. Our study was carried out between May 1^st^ and July 1^st^ 2020, which was concomitant with the peak of SARS-Cov-2 transmission in the city of Santiago. At the start of the pandemic in March, Clinica Indisa had 79 high complexity intensive care unit (ICU) beds. In response to the drastic increase in the transmission of SARS-CoV-2 and in the admission of COVID-19 patients to ICU, the clinic increased its capacity to 172 beds in June (Fig 1A). Albeit this significant increase during the infection peak, the occupancy of these beds remained always close to the limit, reaching 94% of occupancy in June (Fig 1B). This reconversion of beds to ICU also required that different wards in the clinic had to be converted, hence increasing the exposure of HCWs from all different services of the clinic to COVID-19 patients.

**Fig 2:**
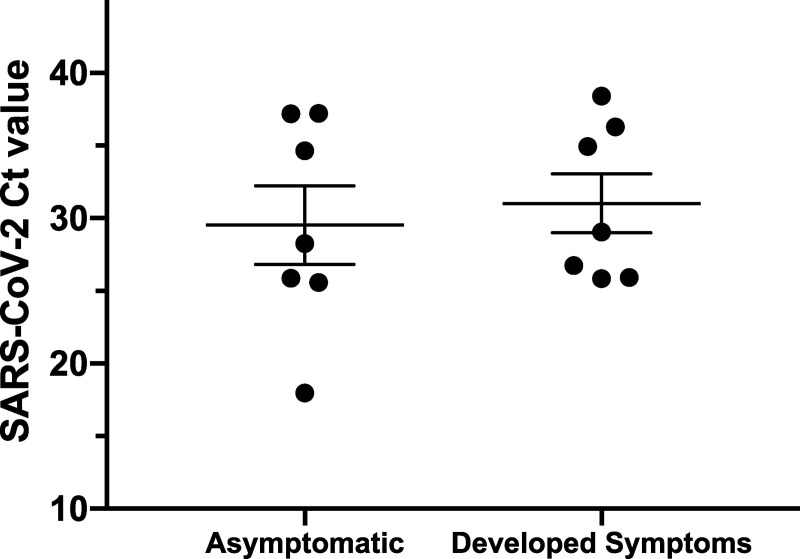
SARS-CoV-2 PCR Ct values of asymptomatic subjects enrolled in the study. Subjects were separated by the complete absence of symptoms or if they developed symptoms within 3 weeks of sample collection. A Ct Value <39 was considered as a positive sample for the presence of SARS-CoV-2.

Subjects were enrolled voluntarily from all services in the clinic. If subjects had previously been infected with SARS-CoV2 they were not able to enroll in the study. Subjects were asked to fill a questionnaire including personal information where they had to detail whether they had presented any COVID-19 symptoms, the presence of any comorbidities and if they had been in direct contact with SARS-CoV-2 infected people. Only volunteers that had not shown any symptoms related to COVDI19 at the moment of sample collection were enrolled in the study. At the same time, an informed consent document was signed. A nasopharyngeal swab was then taken from each individual to pursue the analysis. Our study was conducted to target most of the HCW that had been in contact with COVID-19 patients. Therefore, a total number of 413 asymptomatic HCWs were finally enrolled into the study. Ninety-five point four percent of these HCWs declared that their work had required close physical interaction with patients that may have had COVID-19, that is a distance shorter than 2 meters away. Out of the total individuals enrolled, 75.5% of them were women, with a median age for entire group of 33 years old ([IQR] = 29–40 years) and where only 27.1% claimed to have comorbidities. The study group congregated different professionals in the clinic with a higher representation in the sample of nursing technician (NT) (38.7%) and professional nurses (32.9%) (Table 1). Importantly, all HCWs that were in direct contact with patients suspected of having COVID-19, declared having used the protective elements that are required for their work in these pandemic conditions. From the 413 HCWs that participated in the study, only 14 of them tested positive for the presence of SARS-CoV-2 by RT-qPCR (2 males and 12 females, Table 1), representing this a 3.4% of positivity in asymptomatic HCWs. We found that HCWs working in high-risk areas, where a high rotation of patients occurs, and PPE must be changed constantly such as in Bronchopulmonary ward (BC; 14.3%) and Emergency Services (ES; 12.8%) showed a higher percentage of positive cases (Table 1). Among the positive HCWs the higher proportion of individuals infected were professional nurses and NTs with 6 cases each. Out of the 14 positive cases for SARS-CoV-2, 7 corresponded to HCWs that carried out functions in the ICU, 5 at the emergency service, 1 at bronchopulmonary consultations unit and 1 at the general hospital wards (Table 1). Half of the 14 SARS-CoV-2-positive HCWs developed symptoms within 3 weeks of testing whereas the rest remained asymptomatic. Interestingly, the SARS-CoV-2 PCR Ct values obtained by asymptomatic or individuals that finally developed symptoms did not show any significant differences, suggesting that viral load is not correlated with the development of symptoms. The most common symptoms observed were anosmia (6/7), ageusia and headache (4/7), myalgia (3/7), fever, chest pain, respiratory distress and cough (2/7). Other less frequent symptoms reported were malaise, diarrhea and abdominal pain. Importantly, we did not observe a significant difference between SARS-CoV-2 PCR Ct values in asymptomatic versus pre-symptomatic HCW (Fig 2). From the infected HCWs that were enrolled in our study, none of them had to be admitted at any time in a hospital service.

**Table 1:** Characteristics of HCWs, according to the results of the detection test for SARS-CoV-2.

Characteristics of HCWs (n = 413)		SARS-CoV-2 PCR		p value
		Negative	Positive	
**Sex n (%)**				0.368
	Male	99 (24.8)	2 (14.3)	
	Female	300 (75.2)	12 (85.7)	
	Total	399 (100)	14 (100)	
**Age in years**				
**median (IQR)**				0.810
	Male	34 (29–42)	37 (30–44)	
	Female	33 (28–39)	32 (29–38)	
	Total	33 (29–40)	32 (30–38)	
**Profession n (%)**				0.634
	Nurse	130 (32.5)	6 (42.9)	
	Physiotherapist	19 (4.8)	0 (0)	
	Medical Doctor	11 (2.8)	1 (7.1)	
	Other	71 (17.8)	1 (7.1)	
	AS	14 (3.5)	0 (0)	
	NT	154 (38.6)	6 (42.9)	
	Total	399 (100)	14 (100)	
**Service n (%)**				0.007*
	BC	7 (1.7)	1 (7.1)	
	ES	39 (9.8)	5 (35.7)	
	HW	61 (15.3)	1 (7.1)	
	ICU	292 (73.2)	7 (50.0)	
	Total	399 (100)	14 (100)	
**Comorbidities n (%)**				0,462
	No	292 (73.2)	9 (643)	
	Yes	107 (26.8)	5 (35.7)	
	Total	399 (100)	14 (100)	

AS: Administrative Staff; NT: Higher Level Nursing Technicians; BC: Bronchopulmonary Consultations; ES: Emergency Service; HW: Hospital Wards; ICU: Intensive Care Units (*) = Statistically significant.

**Fig 3:**
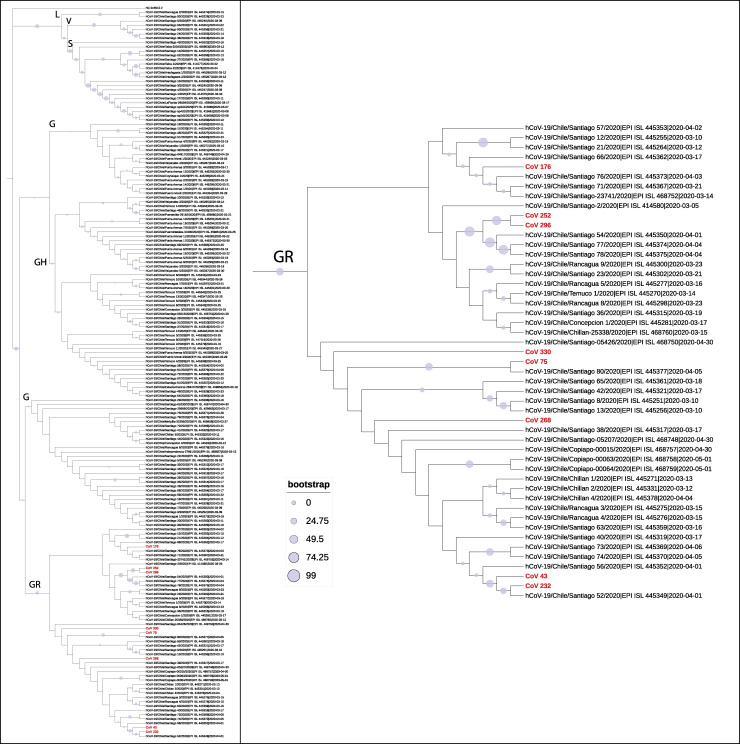
Dendrogram of nucleotide diversity of SARS-Cov-2 genomes isolated and sequenced from samples from asymptomatic Healthcare Workers (HCWs). We constructed a maximum-likelihood tree using 169 full-sequence genomes. Additionally, we included the genome reference NCBI sequence ID NC_045512.2 on the tree. The letters in the tree indicate the lineage to which each clade belongs based on the GISAID classification (L, V, G, GH, and GR;(Han et al., 2019)). The purple circles indicate the bootstrap value in percent for 1,000 iterations. (A) the dendrogram of the 169 full-sequence genomes from Chilean samples (161 full-sequence genomes of SARS-CoV-2 isolated from Chilean patients, which are available in GISAID and eight full-sequence genomes of SARS-CoV-2 isolated from asymptomatic HCWs samples). (B) GR lineage where are located the 8 full-sequence genomes obtained from asymptomatic HCWs samples.

Positive samples from HCWs with Ct value <30, were used for subsequent sequencing of the viral genome. All the samples clustered at the GR GISAID clade (equivalent to clade 20B), as all the genomes presented the polymorphisms C241T, C3037T, A23403G, G28882, S-D614G, and N-G204R (Table 2). GR has been the most frequent clade detected in Chilean cities, including in Santiago. Although some of the sequenced genomes were highly similar, samples Cov-176 and Cov-268 showed numerous differences relative to other genomes in the GR clade (Fig 3). Therefore, these data suggest that there are at least five putative infection sources (Cov-176, Cov-268, Cov-43/Cov-232, Cov-75/Cov-330, Cov-252/Cov-296).

**Table 2:** Details of SARS-CoV-2 positive samples from asymptomatic HCWs.

ID	Asymptomatic	Developed Symptoms	Ct	Sequence	ID seq NCBI	Coverage (X)	Linage
**CoV 43**	yes	yes	26.72	yes	SAMN16255338	100	GR
**CoV 75**	yes	yes	25.89	yes	SAMN16255337	304	GR
CoV 121	yes	yes	35.9	no		-	-
CoV 143	yes	no	34.74	no		-	-
CoV 147	yes	yes	35.20	no		-	-
**CoV 176**	yes	no	28.23	yes	SAMN16255336	163	GR
CoV 186	yes	no	37.24	no		-	-
CoV 193	yes	no	37.47	no		-	-
**CoV 232**	yes	no	25.54	yes	SAMN16255335	161	GR
**CoV 252**	yes	yes	25.63	yes	SAMN16255334	183	GR
**CoV 268**	yes	no	18.11	yes	SAMN16255333	235	GR
**CoV 296**	yes	no	25.88	yes	SAMN16255332	182	GR
**CoV 330**	yes	yes	29.1	yes	SAMN16255331	183	GR
CoV 332	yes	yes	38.4	no		-	-

Samples used for sequencing are highlighted in bold letters.

In summary, these results indicate that only a minor fraction (3.45%) of the HCWs tested from Clinica Indisa were positive for the presence of SARS-CoV-2. Moreover, sequencing of the viral genomes shows that there are at least 5 different infection sources among the infected individuals, supporting the idea that these HCWs became infected at their individual communities outside of the clinic.

## Discussion

Since the beginning of the SARS-CoV-2 pandemic, countries have taken different approaches to stop spreading of the virus. They all coincide at this point that testing and tracing SARS-CoV-2 infected subjects is primordial for slowing down the spread of the virus. However, limited resources and the dependency of diagnostic reagents produced overseas, have diminished the ability of each country to adequately respond to this challenge, especially in Latin America.

It is now known that an elevated proportion of infected people is asymptomatic. Some studies have shown that these individuals infected with SARS-CoV-2 may represent up to 45% of the cases [3]. More importantly, it has been suggested that the asymptomatic population is capable of spreading the virus for more than 14 days without knowing it [2, 3]. Therefore, having a good system in place for tracking and isolating asymptomatic subjects is imperative to control the pandemic as it has been recently demonstrated in Italy [4]. Nevertheless, in most places testing has been mainly focused on symptomatic individuals with only few studies reporting about asymptomatic subjects that are directly exposed to COVID-19. Especially important among this last group are HCWs that are in the front-line attending patients with COVID-19 and that could then represent a hot spot for SARS-CoV-2 transmission in health care institutions.

Our study focused on the identification of asymptomatic HCWs infected with SARS-CoV-2 during the peak of infection rates in Chile. It was found that only 14/413 (3,4%) of HCWs tested positive for SARS-CoV-2 in the absence of symptoms, a significantly small proportion compared to a 9.1% of asymptomatic infections in the country during the time our study was conducted (https://www.gob.cl/coronavirus/cifrasoficiales). Out of these 14 individuals and within 3 weeks after testing, 7 did not develop any symptoms and 7 of them became symptomatic, suggesting that the true asymptomatic carriage rate was 7/413 (1,7%). Our data is comparable to that of recent studies testing HCWs in hospitals of USA and UK, which show asymptomatic infection rates close to 3% [12, 13].

Our study has some limitations, which include the number of enrolled subjects. This was a voluntary study open to all 1379 workers in the clinic, but it was limited by the time of sample collection and HCWs shifts due to law requirements of a mandatory quarantine after a RT-PCR test was run and results were informed. Nevertheless, we were able to reach approximately 30% of the HCWs in the clinic. Our study was conducted in a private clinic in Santiago, Chile. Thus, our results do not necessarily reflect the public hospital system where the use and availability of PPE was limited at the time. At the moment the study was conducted, no testing was performed routinely at either private or public hospitals to detect symptomatic or asymptomatic SARS-CoV-2 HCWs infections. Nowadays in hospitals HCWs are routinely being screened for SARS-CoV-2 infection as part of their protocols.

The higher percentage of positive cases in Bronchopulmonary ward (14.3%) and Emergency Services (12.8%) suggests that these 2 areas are at higher risk of infection than the ICU or general wards. This could be due to the constant changes of PPE that HCWs are required to do between patients, thus at least for these two areas we cannot completely discard nosocomial infection.

Interestingly, the comparison of the SARS-CoV-2 PCR Ct values obtained from asymptomatic individuals and those from individuals that finally developed symptoms, did not show any significant differences. This result suggests that there is no direct relationship between viral load at the moment of testing and the emergence of symptoms associated with COVID-19. As asymptomatic subjects may exhibit elevated viral titers they can be cataloged as “super-spreaders”, capable of silently transmitting the virus among the population to a large number of people [14], our study strongly support the need for systematic testing among asymptomatic HCWs.

Previous studies have shown high nosocomial transmission rates of SARS-CoV-2 between HCWs and/or patients [15]. However, our sequencing results suggest that infection of HCWs occurred in their respective communities, outside of the clinic and was not nosocomial. These results support the strategy of tracing asymptomatic individuals to prevent nosocomial infection among HCWs and patients amidst the COVID-19 pandemic. The broad strategy implemented to prevent SARS-CoV-2 transmission in Clinica Indisa included for all HCWs working in the hospital wards and urgent care working with suspected or confirmed cases of COVID-19, the mandatory use of N95 or KN95 masks, eye protection, a disposable gown with arm coverings and disposable gloves. All HCWs in the clinic were required to alert if any symptoms arose, maintain frequent hand hygiene, avoid any physical contact with colleagues, avoid presential meetings or gatherings, use a protective surgical or N95/KN95 mask at all times covering mouth and nose, maintain proper physical distance of 1 meter or more, regular disinfection of common areas, equipment, desks, telephones, etc. These efforts likely played a major role in avoiding the generation of infection clusters in the clinic facilities due to asymptomatic carriers. These results also provide strong evidence in favor of using appropriate PPE and following established infection control protocols to limit the impact of nosocomial transmission of SARS-CoV-2.
